# Regulation of chemotropic guidance of nerve growth cones by microRNA

**DOI:** 10.1186/1756-6606-4-40

**Published:** 2011-11-03

**Authors:** Liang Han, Zhexing Wen, Rachel C Lynn, Marie-Laure Baudet, Christine E Holt, Yukio Sasaki, Gary J Bassell, James Q Zheng

**Affiliations:** 1 Department of Cell Biology, Emory University School of Medicine, Atlanta, 615 Michael Street, GA 30322, USA; 2 Center for Neurodegenerative Diseases, Emory University School of Medicine, Atlanta, 615 Michael Street, GA 30322, USA; 3 Department of Physiology, Development and Neuroscience, University of Cambridge, Cambridge CB2 3DY, UK; 4 Department of Neurology, Emory University School of Medicine, 615 Michael Street, Atlanta, GA 30322, USA

**Keywords:** Axon guidance, microRNA, translation, BDNF, BMP7, actin cytoskeleton, migration

## Abstract

**Background:**

The small non-coding microRNAs play an important role in development by regulating protein translation, but their involvement in axon guidance is unknown. Here, we investigated the role of microRNA-134 (miR-134) in chemotropic guidance of nerve growth cones.

**Results:**

We found that miR-134 is highly expressed in the neural tube of *Xenopus *embryos. Fluorescent in situ hybridization also showed that miR-134 is enriched in the growth cones of *Xenopus *spinal neurons in culture. Importantly, overexpression of miR-134 mimics or antisense inhibitors blocked protein synthesis (PS)-dependent attractive responses of *Xenopus *growth cones to a gradient of brain-derived neurotrophic factor (BDNF). However, miR-134 mimics or inhibitors had no effect on PS-independent bidirectional responses of *Xenopus *growth cones to bone morphogenic protein 7 (BMP7). Our data further showed that *Xenopus *LIM kinase 1 (Xlimk1) mRNA is a potential target of miR-134 regulation.

**Conclusions:**

These findings demonstrate a role for miR-134 in translation-dependent guidance of nerve growth cones. Different guidance cues may act through distinct signaling pathways to elicit PS-dependent and -independent mechanisms to steer growth cones in response to a wide array of spatiotemporal cues during development.

## Background

Developing axons are guided to their specific targets for complex neuronal connections by a spatiotemporal pattern of extracellular cues [1,2]. The motile tip of the axons, the growth cone, reacts to various guidance molecules with distinct responses, including acceleration of extension, inhibition and/or collapse of growth cones, and turning towards or away from attractive or repulsive cues [2,3]. Recent studies have shown that local protein synthesis and degradation play a role in axon guidance [4-9]. However, the mechanisms underlying protein synthesis (PS)-dependent regulation of growth cone guidance remain to be fully elucidated. In addition to the classical translation mechanism involving the mammalian target of rapamycin (mTOR) [6,10,11], increasing evidence indicates that microRNAs (miRNAs), the non-coding RNAs of ~20-23 bps, regulate mRNA expression [12-14]. MiRNAs often bind to target mRNAs through partial complementary pairing to suppress mRNA translation or decrease mRNA stability and have been shown to participate in the regulation of many, if not all, cellular processes [12-14]. While miRNAs have been shown to play an important role in brain development and functions [15,16], their involvement in axonal growth and guidance remains untested.

In this study, we examined the involvement of miRNAs in growth cone guidance responses of *Xenopus *neurons. We found that the brain specific miR-134 is highly expressed in neural cells of *Xenopus *embryos and abundantly present in the growth cones of embryonic *Xenopus *spinal neurons in culture. To determine the role of miR-134 in growth cone guidance, we performed an in vitro growth cone turning assay and examined growth cone responses to brain-derived neurotrophic factor (BDNF) [8,17-19] and bone morphogenic factor 7 (BMP7) [20,21]. Our data showed that a gradient of BDNF, not of BMP7, depended on PS to steer the growth cone in culture. Interestingly, only BDNF-induced growth cone turning was abolished by miR-134 manipulations, suggesting that miR-134 is selectively involved in PS-dependent guidance responses. Finally, we showed that the 3' untranslated region (3'UTR) of *Xenopus *laevis LIMK1 (Xlimk1) mRNA could be a potential target for miR-134 binding and regulation. Together, these results support a role for miRNAs in regulation of selected guidance responses of nerve growth cones.

## Methods

### *Xenopus *embryo injection and cell culture

Blastomere injection of miR-134 mimics or antisense inhibitors (RNA oligonucleotides, 20 μM, Thermo Scientific, catalog numbers: C-300628-05 and IH-300628-06) into *Xenopus *embryo was preformed as described previously [22]. Typically, 2-10 nl of the oligonucleotides (control, mimic, or antisense) were microinjected into one blastomere of *Xenopus *embryos at one-cell or two-cell stage, together with fixable FITC-dextran (10 mg/ml, Invitrogen) as the fluorescent tracer. Embryonic *Xenopus *spinal neurons were then isolated from stage 20-22 *Xenopus *embryos and cultured on glass coverslips that were pre-coated with poly-d-lysine and laminin as described previously [22]. The cultures were kept at 20-22°C in a serum-free medium (SFM) containing of the following: 50% (v/v) Leibovitz L-15 medium (Invitrogen), 50% (v/v) Ringer's solution (115 mM KCl, 2 mM CaCl_2_, 2.6 mM KCl, 10 mM HEPES, pH 7.4), and 1% (w/v) BSA (Sigma). Neurons with the fluorescence of FITC-dextran were identified and used for experiments. All the experiments involving *Xenopus *frogs and embryos were carried out in accordance to the NIH guideline for animal use and have been approved by the institutional animal care and use committee (IACUC) of Emory University.

### Growth cone turning induced by extracellular guidance gradients

Growth cone turning induced by BMP7 or BDNF gradients was performed according to the method described previously [22]. For BDNF-induced turning, cells were used 6-12 hr after plating. For BMP7 turning assays, *Xenopus *neurons cultured for 4-8 hr (young) and 20-24 hr (old) were tested for attraction and repulsion, respectively. The concentration gradients of the guidance molecules were created by pulsatile repetitive pressure ejection of the solution through a glass micropipette (1 μm opening, repetitive rate at 2 Hz, air pressure at 3 psi, 100 μm away from the growth cone with a 45 degree angle). The turning assay was performed on a Nikon TE300 microscope equipped with a 20× NA 0.45 dry objective. The digital images of the growth cone at the onset and the end of the 30 min assay were acquired by a SensiCam CCD camera (Cooke Scientific). The images were then overlaid with pixel-to-pixel accuracy, and the trajectory of new neurite extension was traced using Adobe Photoshop (Adobe Systems). The turning angle was defined as the angle between the original direction of neurite extension and a line connecting the positions of the growth cone at the experiment onset and at the end of 30 min exposure to the gradient. Neurite extension was quantified by measuring the entire trajectory of net neurite extension over the 30 min period. Only growth cones extending 5 μm or more were scored for turning responses. The nonparametric Mann-Whitney test was used to analyze turning angles. Recombinant human BDNF (50 μg/ml in pipette) was purchased from Peprotech (Rocky Hill, NJ). Recombinant human BMP7 (5 μM in pipette) was purchased from R&D Systems (Minneapolis, MN). Cycloheximide (25 μM) was purchased from Calbiochem (San Diego, CA) and added to bath 20 min before the onset of BDNF or BMP7 gradients.

### Fluorescence and whole mount in situ hybridization

Fluorescence in situ hybridization (FISH) with digoxigenin-labeled probes was performed to visualize the presence of Xlimk1 mRNA and miR-134 in growth cones. Three different modified oligonucleotides (50 bases each) complementary to the coding sequence of Xlimk1 mRNA were purchased from Biosearch Technologies (CA). Each oligonucleotide was modified at five positions within the sequence and chemically labeled using digoxigenin succinamide ester (Boehringer Mannheim). Reversed probes were used as negative controls. After hybridization for the Xlimk1 mRNA, the probes labeled with digoxigenin were detected using Cy3-conjugated monoclonal antibody (mAb) to digoxigenin and anti-mouse mAb-Cy3 (Jackson ImmunoResearch Labs).

FISH detection of miR-134 was performed using locked nucleic acid (LNA) modified probes [23,24]. Digoxigenin-labeled LNA probes for mature miR-134 were purchased from Exiqon (Denmark). Scramble digoxigenin-labeled LNA probes were used as the negative control. For whole mount in situ hybridization (ISH), after hybridization, monoclonal antibody to digoxigenin conjugated to horseradish peroxidase (HRP, Roche) was used and the embryos were incubated in NCIP/NBT substrate to visualize the signals. For FISH on cell cultures, monoclonal antibody to digoxigenin conjugated to HRP (Roche) was used and the fluorescence signals were generated by the tyramide amplification system (PerkinElmer). Images were acquired using a Nikon C1 laser scanning confocal system on a Nikon microscope. For each cell, a confocal z-stack of the growth cone (~10 optical slices) was acquired using a small pinhole. The z-stack was then collapsed by maximal intensity projection to generate the 2-D image.

In the double FISH assay, FITC-conjugated LNA-miR-134 probes and digoxigenin-conjugated Xlimk1 probes (Biosearch) were co-hybridized on cultured neurons overnight. The fluorescent signals were generated sequentially; first miR-134 was detected with anti-fluorescein-HRP (Perkin Elmer) followed by amplification with the fluorescein tyramide signal amplification (TSA) system (Perkin Elmer). After inactivation of HRP with 3% H_2_O_2_, Xlimk1 signals were detected following the same procedure with anti-digoxigenin-HRP (Roche) followed by amplification with Cy3-TSA.

### MicroRNA expression analysis

MirVana™ miRNA Isolation Kit (Applied Biosystems) was used to isolate small RNAs enriched in microRNAs from rat brain, which is used as the positive control for PCR analysis. Total RNAs were collected from stage 20-22 *Xenopus *whole embryos or neural tube tissues. The first strand cDNA for PCR were synthesized using SuperScript™ III First-Strand Synthesis System (Invitrogen). Taqman stem-loop real-time PCR assays (Applied Biosystems) were used to detect the expression of mature microRNAs and Ct values (the PCR cycle number to reach the fluorescence threshold) were analyzed with SDS software (Applied Biosystems) and normalized to the expression level of β-actin.

### Luciferase assay

The *Xenopus *laevis Limk1 3' UTR was amplified by 3' RACE System for Rapid Amplification of cDNA Ends (Invitrogen) from stage 22 *Xenopus *laevis embryo cDNA. PCR products were sequenced and the results were submitted to NCBI [GenBank: GU227145]. The 3'UTR of Xlimk1 mRNA was fused downstream to the luciferase reporter. The mRNAs of luciferase-Xlimk1 3'UTR and Renilla were prepared using mMessenger Machine in vitro transcription kit (Ambion) and were injected into *Xenopus *embryos together with miR-134 mimics or control oligonucleotides (luciferase-Xlimk1 3'UTR mRNA 100 ng/μl, Renilla mRNA 10 ng/μl, microRNA mimic and control oligonucleotide 20 μM). Embryos were lysed 2 hr after microinjection and the luciferase activity was measured using Dual-Luciferase Reporter Assay System (Promega).

### Immunofluorescent staining and quantification

*Xenopus *cell cultures were prepared from embryos injected with or without miR-134 mimics or inhibitors together with the fixable FITC-dextran. *Xenopus *neurons (6-12 hr after plating) were bath exposed to BDNF (50 ng/ml) or control Ringers' solution for 30 min before they were rapidly fixed. The cells were fixed with 4% paraformaldehyde in a cacodylate buffer (0.1 M sodium cacodylate, 0.1 M sucrose, pH 7.4) for 30 min, washed three times in 100% Ringer's saline, and permeabilized with Triton X-100 (0.1%) for 10 min. The cells were first incubated with 5% goat serum to block nonspecific binding sites for 1 hr at room temperature. The cells were then incubated with a rabbit antibody against phospho-p44/42 (Thr202/Tyr204) (Cell Signaling, Danvers, MA) or against LIMK1 (Novus, Littleton, CO) overnight at 4°C. Afterwards, the cells were labeled by Cy3-conjugated goat anti-rabbit secondary antibodies (Jackson Lab). *Xenopus *neurons injected with miR-134 mimics or antisense inhibitors were identified by their FITC-dextran fluorescence.

Fluorescent imaging was performed on a Nikon inverted microscope (TE2000) using a 60× N.A. 1.4 Plan Apo objective. Digital images were acquired by a SensiCam QE CCD camera (Cooke Scientific) through the use of IPLab software (BD Biosciences). To quantitatively determine the immunofluorescence of phospho-p44/42 and its changes in response to BDNF, we maintained the same imaging settings for each batch of samples containing control and treated cells. Background subtracted images were analyzed by creating a region of interest (ROI) that circumscribed the growth cone using ImageJ software (NIH). For each growth cone, the ROI intensity was normalized to the average from its peer control (without BDNF treatment). Data for each condition were from at least two separate batches of *Xenopus *cultures on different days.

## Results

**Figure 1:**
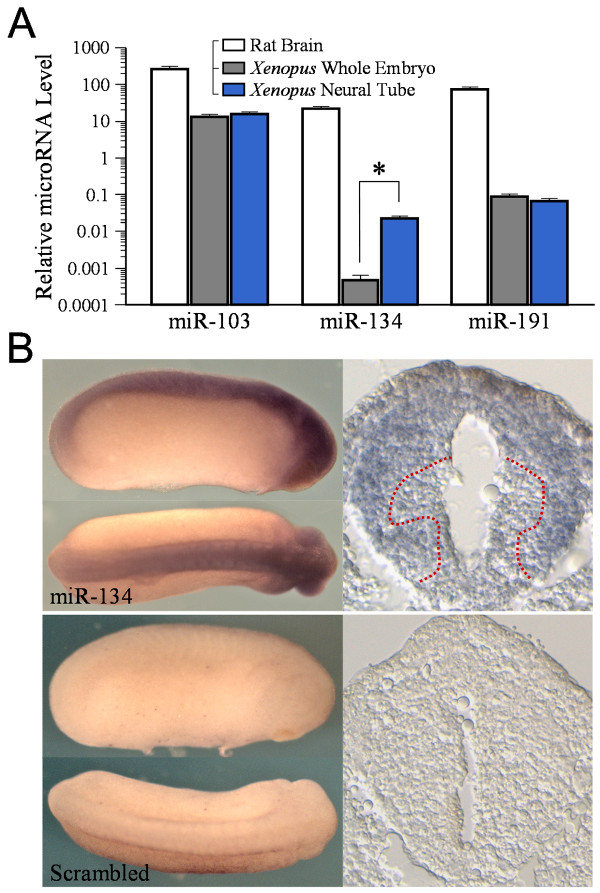
**Presence of miR-134 in *Xenopus *neural tissues**. (**A**) Taqman stem-loop real-time PCR assays on the expression of three miRNAs, miR-103, miR-134, and miR-191 in rat brain, whole *Xenopus *embryos, and *Xenopus *neural tube tissues. Please note that both *Xenopus *samples were total RNAs, whereas the rat brain sample was processed for microRNA enrichment using a mirVana miRNA isolation kit (see Methods). As a result, the microRNA level in the rat brain sample was much higher and merely serves as a positive control. The relative miRNA levels are plotted in logarithmic scale. The error bars represent the standard error of the mean. Asterisk: p < 0.01 Student's *t*-test. (**B**) Whole mount in situ analysis of miR-134 expression in *Xenopus *embryos (stage 24) using a LNA probe against miR-134 or a scrambled probe. Cross sections of the spinal cord are shown on the right side. Dotted red lines depict the boundary of miR-134 positive and negative regions.

Among many brain-enriched miRNAs, miR-134 was shown to regulate LIM kinase 1 (LIMK1) translation in dendritic spine plasticity [16]. Given the important role for LIMK1 and its downstream target ADF/cofilin in growth cone motility and guidance [22,25], we examined if miR-134 regulates guidance responses of *Xenopus *growth cones. We first performed Taqman stem-loop real-time PCR to examine the expression of three mature miRNAs, miR-103, miR-134, and miR-191, in *Xenopus *embryos. All three microRNAs have been shown to be expressed in mammalian brains [26]. We found that these three microRNAs are expressed in *Xenopus *whole embryos and neural tubes, as well as in rat brain tissues (Figure 1A). In particular, the expression level of miR-134 appears to be much higher in *Xenopus *neural tube than that in the other parts of the embryo, suggesting preferential expression of miR-134 in the central nervous system. We next performed whole-mount in situ hybridization in *Xenopus *embryos using LNA probes against miR-134 [23,24]. Consistent with the PCR data, we detected a high level of miR-134 in the brain, retina and dorsal neural tube regions of stage 24 *Xenopus *embryos (Figure 1B). Cross sections of the spinal cord showed that miR-134 is present in the dorsal, mid and lateral regions where commissural axons and motor neurons reside, but not in or around the ventral midline (Figure 1B, dashed lines depicting the approximate boundary between labeled/unlabeled regions). On the other hand, no signal was detected using a scrambled probe. Therefore, miR-134 is expressed in the nervous system of *Xenopus *embryos at a developmental stage involving axonal elongation and guidance [27,28].

**Figure 2:**
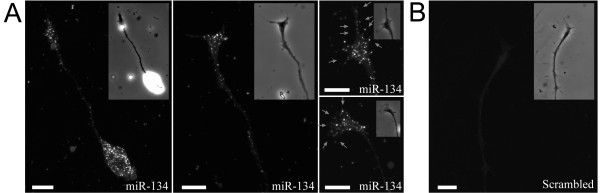
**Enrichment of miR-134 in *Xenopus *growth cones**. Fluorescence in situ hybridization was used to detect miR-134 in cultured *Xenopus *spinal neurons using a LNA probe (A) or scrambled probe (B). Phase contrast images of the growth cones are shown as insets. Arrows indicate the miR-134 puncta in the lamellipodia and filopodia. Scale bars: 10 μm.

We next investigated the subcellular distribution of miR-134 in embryonic *Xenopus *neurons in culture by FISH. While scrambled control probes produced a very low level of background signals (Figure 2B), miR-134-specific LNA probes revealed a high level of miR-134 in the cell body and, importantly, the distal growth cones (Figure 2A). Interestingly, some of the miR-134 signals were seen at the periphery of the growth cone, including the actin-rich lamellipodia and filopodia (arrows). This pattern of miR-134 localization in the growth cone has been observed for almost all the cells examined (> 50 cells), suggesting a potential function for miR-134 in growth cone migration and guidance. The enrichment of miR-134 in growth cones also suggests that miR-134 may be actively localized to and/or locally produced in the distal axonal compartments [29].

**Figure 3:**
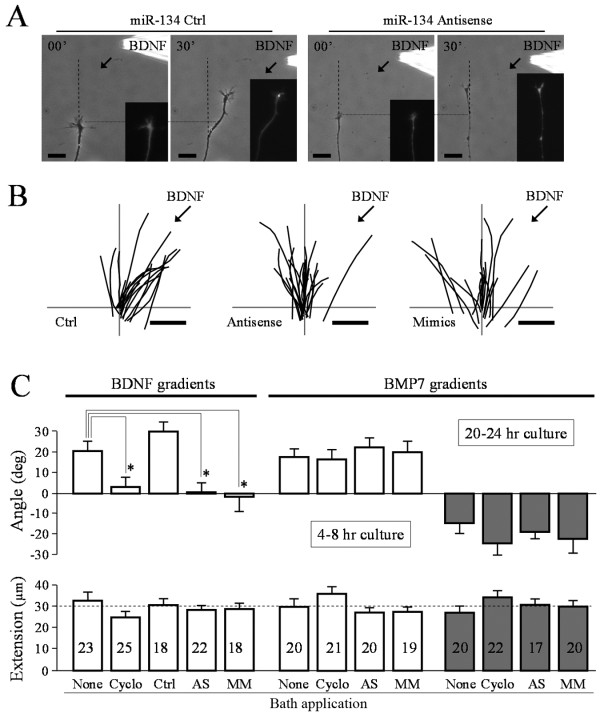
**Blockade of BDNF-induced growth cone turning by miR-134 mimic and antisense oligonucleotides**. (**A**) Representative images of growth cones at the beginning and the end of 30 min exposure to a BDNF gradient. Dashed lines indicate the original direction of growth cone extension. Dotted lines indicate the position of the growth cone at the onset of the turning assay. Insets are fluorescent images of the same growth cones, showing the presence of the fluorescent tracer FITC-dextran co-injected with miR-134 mimic or antisense. (**B**) Trajectory tracings of all the growth cones subjected to 30 min turning assay in a BDNF gradient. Neurons injected with control oligonucleotides (Ctrl), miR-134 antisense inhibitors or mimics are shown here. The origin is the center of the growth cone at the onset of the BDNF gradient and the original direction of growth cone extension is vertical. Scale bars: 20 μm. Arrows indicate the direction of the BDNF gradient. (**C**) Average turning angles (top) and net extension (bottom) of different groups of all the growth cones examined. Numbers indicate the total number of growth cones examined for each group. Each group is labeled at the bottom: None: neurons not treated; Cyclo: bath application of cycloheximide; Ctrl: neurons injected with miR-134 control oligonucleotides; AS: injected with miR-134 antisense inhibitors; MM: injected with miR-134 mimics. Error bars represent the standard error of the mean. Asterisks depict the statistical significance (p < 0.01, Mann-Whitney test).

To evaluate a potential role for miR-134 in growth cone guidance, we performed in vitro turning assays to examine PS-dependent growth cone responses to a BDNF gradient [8,18,19], in conjunction with overexpression of synthetic miR-134 mimics or antisense inhibitors. These miRNA mimics are designed to enter the miRNA pathway to act as mature miRNA whereas miRNA antisense oligonucleotides specifically target and irreversibly bind endogenous miRNA. Both approaches have been successfully used to interfere with endogenous miRNA functions [30-32]. Consistent with previous studies [8,33], BDNF gradients (50 μg/ml in pipette, about 50 ng/ml reaching the growth cone) elicited marked attractive turning of *Xenopus *growth cones cultured on laminin substrate, which was not affected by overexpression of a control oligonucleotide (Figure 3A). The attractive response is better depicted by the tracings of growth cone extension of all the neurons exposed to 30 min of BDNF gradients (Figure 3B), as a majority of the growth cones extended towards the BDNF source. However, overexpression of miR-134 antisense inhibitors or mimics completely blocked the turning response to BDNF (Figure 3A&B). Quantitative analysis confirmed that BDNF-induced attraction was completely abolished by miR-134 antisense inhibitors and mimics (Figure 3C). Application of the PS inhibitor cycloheximide (25 μM) also blocked growth cone attraction to BDNF, confirming its PS-dependence (Figure 3C).

**Figure 4:**
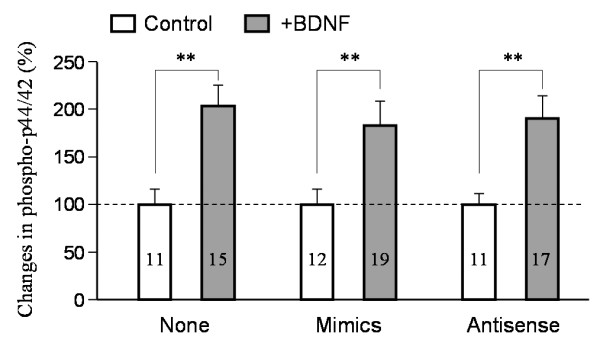
**Quantitative analysis of BDNF-induced p44/42 MAPK activation by immunofluorescence of phospho-p44/42 levels in *Xenopus *growth cones**. Neurons injected with control, miR-134 mimics and antisense inhibitors were exposed to control saline or BDNF (50 ng/ml) for 30 min. The immunofluorescence intensity of BDNF-exposed growth cones was normalized to that of the corresponding group without BDNF exposure. Numbers indicate the number of growth cones examined. Error bars: the standard error of the mean. Double asterisks: p < 0.001 (Student's *t*-test).

We next examined the growth cone response to another guidance cue BMP7 [20,21]. We previously showed that a gradient of BMP7 can elicit bidirectional turning responses: attraction in young neurons (4-8 hr in culture) and repulsion in relatively mature neurons (20-24 hr in culture) [22]. We first tested if BMP7-induced growth cone turning depends on PS. Bath application of cycloheximide did not affect either attraction or repulsion in response to BMP7 gradients (Figure 3C). Importantly, neither attraction nor repulsion induced by BMP7 was affected by miR-134 antisense inhibitors or mimics (Figure 3C). To determine if miR-134 mimics or antisense inhibitors disrupted BDNF signaling in general, we examined the phosphorylation level of p44/42, the mitogen-activating protein kinase that is known to be activated by BDNF [34]. We found that miR-134 mimics or antisense inhibitors had no effect on p44/42 activation by BDNF as evidenced by a similar level of increase in phospho-p44/42 (Thr202/Tyr204) in response to BDNF (Figure 4). Together, these results indicate that miR-134 is likely involved in translation-dependent BDNF guidance responses of *Xenopus *growth cones.

**Figure 5:**
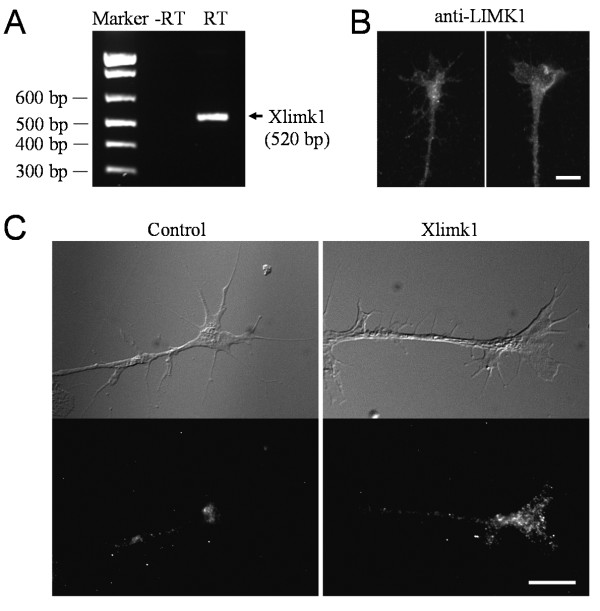
**Detection of *Xenopus *limk1 in *Xenopus *neurons**. (**A**) RT-PCR detection of Xlimk1 mRNA from RNA samples extracted from Stage 20-22 *Xenopus *neural tube tissues using specific primers. RNA samples were processed without (-RT) and with reverse transcriptase (RT). (**B**) Representative fluorescence images of cultured *Xenopus *growth cones labeled using a specific antibody against LIMK1. (**C**) Detection of Xlimk1 mRNA in *Xenopus *growth cones by fluorescence in situ hybridization. Top panels are the differential interference contrast (DIC) images of the growth cones. Bottom panels are the FISH images of *Xenopus *growth cones labeled with digoxigenin-conjugated probes (three probes, ~50 nt each) that are specifically complementary to different parts of the coding region of Xlimk1 mRNA. The reverse probes were used as the control. Scale: 10 μm.

**Figure 6:**
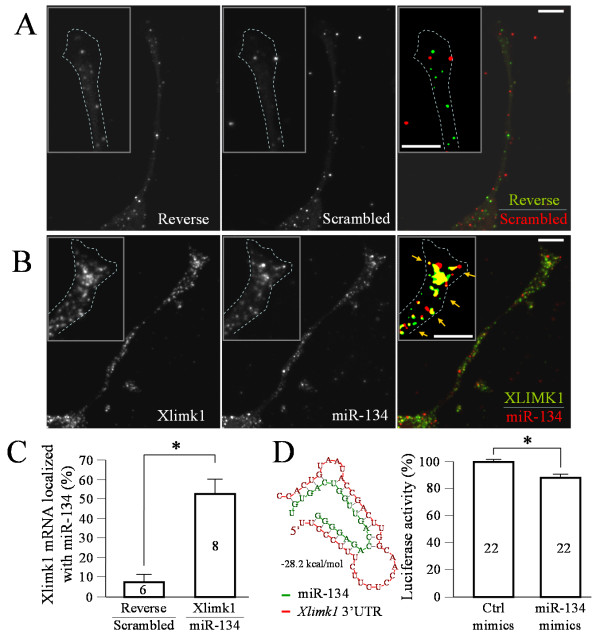
***Xenopus *limk1 mRNA as a potential target of miR-134**. (**A-B**) Representative confocal double FISH images showing growth cones subjected to either reverse Xlimk1 probes and scrambled miRNA probes (A) or Xlimk1 probes and LNA-miR-134 probes (B). Color panels are the merged channels of both Xlimk1 (green) and miR-134 (red) signals, of which an intensity threshold was applied to each channel to highlight the FISH signals. Yellow colors indicate co-localization of Xlimk1 and miR-134. Scale bars: 10 μm. (**C**) Quantification of the percentage of Xlimk1 puncta co-localized with miR-134 puncta. Numbers indicate the number of growth cones examined. (**D**) The predicted duplex formation between miR-134 (red) and Xlimk1 (green) 3'UTR is shown on the left (optimal binding energy: -28.2 kcal/ml). The results of luciferase assays using a Xlimk1 3'UTR-luciferase reporter are shown in the bar graph on the right. Numbers indicate the total numbers of samples from three repeated trials. Error bars depict the standard error of the mean. Asterisks in (C & D): p < 0.01 Student's *t*-test.

Since miR-134 was shown to regulate LIMK1 translation in hippocampal neurons [16], we suspected that *Xenopus *limk1 mRNA could be a potential target of miR-134 regulation. We first confirmed the presence of *Xenopus *limk1 (Xlimk1) mRNA in *Xenopus *neural tubes by RT-PCR (Figure 5A). Immunostaining also showed the presence of LIMK1 in *Xenopus *growth cones (Figure 5B). Importantly, FISH detected an enrichment of Xlimk1 mRNA in the growth cone (Figure 5C). To test if *Xenopus *Limk1 mRNA could be a target of miR-134, we first performed double FISH. We found that both Xlimk1 mRNA and miR-134 are highly expressed in *Xenopus *growth cones in culture, seen as fluorescent puncta (Figure 6B). Significantly, a large percentage of Xlimk1 mRNA puncta were co-localized with miR-134 puncta (color panel in Figure 6B; arrows), whereas the control generated a low level of signals without colocalization (Figure 6A). Quantitative analysis showed that about 50% of Xlimk1 mRNA puncta were colocalized with miR-134 FISH signals, whereas less than 10% colocalization was observed for the control (Figure 6C). MiRNAs function by directing mRNA degradation or disrupting mRNA translation mostly through partial complementary pairing with the 3' untranslated region of target mRNAs [12,13]. Since the 3' UTR of *Xenopus *laevis Limk1 mRNA was not published, we cloned the 3' UTR of Xlimk1 [GenBank: GU227145] and found a potential miR-134 binding site (Figure 6D). We next constructed a luciferase reporter linked to Xlimk1 3'UTR and performed the luciferase assay. We found that miR-134 mimics, but not the control oligonucleotide, was able to significantly reduce the luciferase expression level (~ 12% reduction in average; Figure 6D). Therefore, Xlimk1 mRNA is a potential target of miR-134 in *Xenopus *neurons.

## Discussion

Our findings represent, arguably, the first evidence for the involvement of miRNAs in regulation of growth cone guidance responses. The presence of miR-134 in the neural tissues of developing *Xenopus *embryos and its localization in motile growth cones indicate a possible role for miR-134 in axonal development. The involvement of miR-134 in guidance responses is best supported by the findings that overexpression of miR-134 mimics or antisense inhibitors selectively abolished PS-dependent attractive responses of the growth cones to BDNF gradients. In synaptically-connected hippocampal cultures, miR-134 was shown to localize in dendritic spines to negatively regulate the translation of LIMK1, a key upstream regulator of ADF/cofilin family of actin regulatory proteins [16]. BDNF was found to relieve miR-134 inhibition of LIMK1 local translation, thus promoting actin polymerization and spine enlargement during synaptic plasticity. It is thus possible that miR-134 could function similarly in *Xenopus *growth cones to regulate LIMK1 translation and actin dynamics. We indeed identified a potential binding site of miR-134 in the 3'UTR of Xlimk1 mRNA. Importantly, double FISH detection also found that a substantial number of Xlimk1 mRNA puncta localized with miR-134 signals in *Xenopus *growth cones. Moreover, we found that miR-134 mimics significantly reduced Xlimk1 3'UTR-luciferase reporter expression, demonstrating that miR-134 can indeed suppress Xlimk1 translation. While the reduction in luciferase expression in our Xlimk1 3'UTR-luciferase assay was relatively small, it was statistically significant in comparison to the control group (Figure 6D). It should be noted that our luciferase assays were performed using the whole embryos at the 1-2 blastomere stage for the ease of microinjection and the large cytoplasmic volume. A better way for assessing miR-134 effects on Xlimk1 translation requires the expression of reporters and assay of their activity in a relatively pure *Xenopus *neuronal population, an experimental system that is unfortunately not available at this moment. Nonetheless, the impact of miR-134 on Xlimk1 translation, although small, could have a major impact on growth cone turning as it might be sufficient in establishing a small asymmetry in Xlimk1 translation to modulate actin dynamics for growth cone steering [35,36]. Moreover, each miRNA typically has several target mRNAs and Xlimk1 mRNA could be one of the many mRNAs targeted by miR-134 in growth cones turning responses. For example, it was shown that miR-134 can target additional mRNAs, including the mRNA encoding the translational repressor Pumilio2 [37]. Clearly, future experiments to identify additional target mRNAs of miR-134 involved in growth cone guidance are needed.

**Figure 7:**
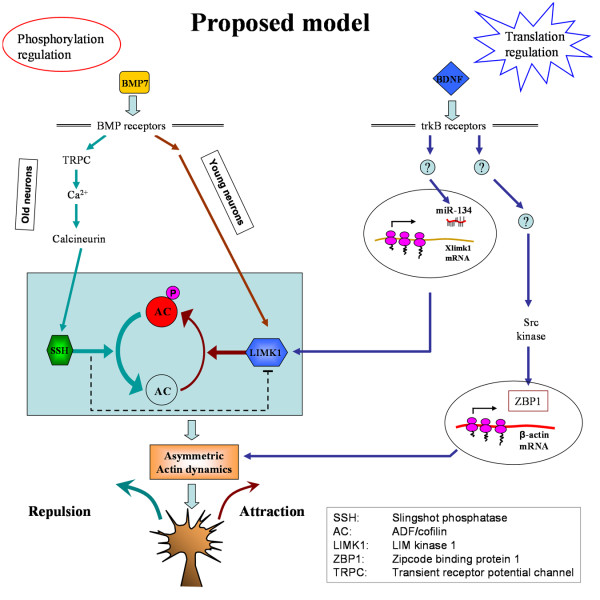
**The schematic diagram shows a proposed model on how different cues might act through PS-dependent and -independent pathways to regulate growth cone steering**. BMP7 acts as bidirectional guidance molecular through phosphorylation regulation of actin depolymerizing factor (ADF)/cofilin (AC). We propose that BDNF gradients release the inhibition of translation by miR-134 and induce the local translation of Xlimk1 in an asymmetric way, leading to the asymmetric modification of the actin dynamics for growth cone steering. BDNF also acts to elicit asymmetric β-actin translation, which could plausibly operate in parallel to or in concert with the miR-134 regulation of LIMK1 translation for growth cone turning. Future experiments are required to test this model. For simplicity, the mTOR pathway is not depicted in the model.

BDNF-induced growth cone turning has been shown to depend on local PS, especially that of β-actin. While the canonical mTOR translation pathway regulates β-actin translation, the zipcode binding protein ZBP1 and its *Xenopus *homolog vgRBP are believed to bind β-actin mRNA and suppress its translation during transport to the final destination [4,8,38]. A BDNF gradient appears to induce asymmetric distribution and translation of β-actin mRNA for growth cone turning. The involvement of miR-134 in BDNF guidance observed in this study adds an additional level of regulation in terms of local mRNA translation. The potential involvement of LIMK1 translation and its regulation by miR-134 could operate in a synergistic way with asymmetric β-actin synthesis for growth cone steering (see Figure 7 for the model). The fact that both miR-134 mimics and antisense inhibitors abolished BDNF-induced turning responses without affecting the neurite extension suggests that miR-134 may be primarily involved in creating or regulating BDNF-induced asymmetry in actin dynamics during steering. It is conceivable that a concentration gradient of BDNF could elicit asymmetric production of both β-actin (through Src and ZBP1) and LIMK1 (through miR-134) across the growth cone, leading to asymmetric actin polymerization for attractive growth cone turning (Figure 7). The presence of miR-134 antisense inhibitors will likely attenuate miR-134 regulation of Xlimk1 mRNA translation, thus abolishing BDNF-induced turning responses. While miR-134 mimics will result in an increase in the miR-134 level, its regulation of Xlimk1 translation could, in principle, still be regulated by BDNF. However, the finding that miR-134 mimics also blocked BDNF-induced turning responses suggests that the excessive amount of miR-134 may have overwhelmed the BDNF regulation, leading to the attenuation of the asymmetric signaling needed for directional responses of the growth cone.

It was striking to see that similar manipulations of miR-134 produced no effects on the bidirectional turning responses induced by BMP7 gradients. Our previous study showed that BMP7-induced bidirectional growth cone turning is mediated by phosphorylation regulation of ADF/cofilin activity through a balancing act of LIMK1 and Slingshot phosphatase (SSH) [22]. ADF/cofilin is inhibited through phosphorylation of its serine 3 residue by LIMK1 and activated through dephosphorylation by SSH [39]. BMP7 appears to act through distinct signaling pathways to activate either LIMK1 or SSH for attractive or repulsive turning responses, respectively, in neuronal cultures with different ages [22]. Importantly, we have found that BMP7-induced bidirectional responses were PS-independent. In this case, the baseline level of LIMK1 and other molecules under PS inhibition could be sufficient for phosphorylation-dependent signal transduction and asymmetric modification of the actin dynamics for growth cone steering. Finally, the inability of miR-134 mimics and antisense inhibitors to influence BMP7-induced bidirectional turning highlights two significant points. First, the effects of miR-134 on BDNF-induced turning are likely specific and not a result of general disruption of growth cone steering. Second, BDNF and BMP7 gradients appear to elicit distinct translation- and phosphorylation-dependent pathways that converge on ADF/cofilin to regulate asymmetric actin dynamics for directional growth cone steering (Figure 7). Conceptually, such a model could provide an effective and flexible mechanism for developing axons to respond to a large and diverse number of guidance cues. It is conceivable that a myriad of signaling cascades could be elicited by these extracellular cues to target protein phosphorylation and/or translation; the convergence of these pathways on a common set of downstream effectors controlling the actin cytoskeleton will enable the growth cone to effectively respond with distinct motile behaviors.

## Competing interests

The authors declare that they have no competing interests.

## Authors' contributions

LH, ZW, RCL contributed equally to this project. As a part of her PhD thesis work, LH initiated the project and performed most of the experiments on the presence of miR-134 in *Xenopus *neurons and growth cones. ZW did the turning assays and RCL cloned the 3'UTR of *Xenopus *laevis Limk1 and performed most of the molecular cloning and FISH experiments. MLB performed the whole mount in situ detection of miR-134 in *Xenopus *embryos and CEH provided the feedback on the miR-134 experiments. YS did the initial Xlimk1 FISH and GJB provided the input to the experiments. JQZ designed and planned the project, provided guidance to the project, performed some of the imaging experiments, and wrote the paper with LH. All authors have read and approved the final manuscript.
